# High-Precision Interferometric Measurements of Gas Refractive Index Using Homodyne Detection

**DOI:** 10.3390/s25113519

**Published:** 2025-06-03

**Authors:** Yanan Miao, Fang Xie, Wentao Feng, Yifeng Zhu, Xun Zhang, Fang Liu

**Affiliations:** Department of Physics, Nanjing Tech University, Nanjing 211816, China

**Keywords:** homodyne detection, Mach–Zehnder interferometry, gas sensing, air refractive index

## Abstract

Balanced homodyne detection, which offers advantages that include low noise and strong anti-interference capabilities, is commonly used as a detection method in quantum metrology. In this article, we propose application of the balanced homodyne detection technique to the gas sensing and measurement field. By constructing a Mach–Zehnder interferometer based on balanced homodyne detection, we realize high-precision measurement of the refractive index of air. The device exhibits interference efficiency of 99% and a common-mode rejection ratio of 40 dB, thus enabling dynamic monitoring of optical phase changes. Under conditions that include a stabilized temperature of 25 °C, atmospheric pressure of 100.08 kPa, and relative humidity of 30%, the refractive index of air was measured experimentally to be n=1.0002711 with a measured minimum standard deviation of $1\times 10^{-7}$. The proposed technique provides high measurement sensitivity and stability, and it also offers the advantage of noncontact measurement. Furthermore, the proposed scheme is applicable to both measurement and dynamic sensing of the refractive indices of gases, along with sensing and measurement of transparent liquids and biological samples.

## 1. Introduction

High-precision gas refractive index sensing is indispensable for atmospheric monitoring, industrial process control, and biomedical diagnostics applications [1,2,3]. In atmospheric monitoring, accurate measurements of refractive index fluctuations are essential for air quality modeling, tracking of greenhouse gas distributions, and improved precision in weather forecasting and climate change studies. In industrial process control, refractive index sensing ensures real-time monitoring of the purity and composition of gases for the use in high-precision manufacturing environments. In biomedical diagnostics, gas refractive index sensing offers a non-invasive detection method for physiological biomarkers contained in exhaled breath or bodily fluids.

Interference-based measurement is characterized by advantages such as high sensitivity, noncontact measurement, robust anti-interference properties, and a real-time monitoring capability, and has emerged as a core technique for analysis of gas refractive indices in atmospheric environmental monitoring. Interferometers, including the Michelson, Mach–Zehnder, and Sagnac types, are used widely to perform precise measurements of a variety of parameters, including light wavelength, temperature, refractive index, pressure, crystal thickness, and solution concentrations [4,5,6,7,8]. Among these devices, the Mach–Zehnder interferometer (MZI) is used prominently in precision quantum measurements because of advantageous characteristics such as its simple structure, ease of integration, high stability, high sensitivity, and low optical loss [9,10,11]. The MZI is commonly used in optical phase estimation, with the first demonstration of optical phase measurements that surpassed the classical shot noise limit being achieved using an MZI [12]. Recently, Qin et al. developed an MZI sensor using four-core sapphire-derived optical fibers that enabled simultaneous measurement of high temperature and high strain [13]. Shaheen et al. measured the refractive index of air at different pressures and temperatures using an MZI [14], while Yu et al. proposed a direct object recovery method based on an MZI structure, thus providing a new optical imaging and diffraction analysis tool [15]. In the gas sensing and measurement field, Tang et al. designed plasmonic sensors based on metal–insulator–metal (MIM) waveguides and tunable MIM waveguide systems and achieved highly sensitive gas measurements with both sensor types [16,17]. Despite these achievements, the existing methods face critical limitations. For example, vacuum extraction techniques involve strict environmental requirements [5,14]. Quantum light source-dependent approaches are both costly and unstable [9], and some of these methods rely on complex manufacturing processes [16,17]. To address these challenges, this paper uses a free-space MZI to perform all experimentation. This configuration offers several distinct advantages: a simple structure, a noncontact measurement capability, cost-effectiveness, and suitability for use in sensor-based measurements.

In precision measurements, the selection of the detection technique has a major impact on the system’s signal-to-noise ratio (SNR). Fiber optic interferometer-based sensing often faces limitations: the technique is restricted by the spectrometer’s resolution, it exhibits poor resistance to environmental interference, and it requires complex melting-based manufacturing processes [6,11,13]. In addition, charge-coupled device-based detection suffers from insufficient sensitivity for weak signals [14,15]. In contrast, balanced homodyne detection (BHD), an important quantum state detection method, offers the advantages of low noise and a high SNR, which are particularly helpful for weak signal detection [18,19,20]. Currently, the BHD technique is applied extensively in quantum precision measurements. Wang et al. performed an in-depth analysis of the unique role played by the homodyne detection mechanism in quantum randomness expansion [21]. Yan et al. used BHD to improve the SNR of a rotating Doppler signal, thus greatly promoting engineering applications of the rotating Doppler effect [22]. Wang et al. developed a low-noise BHD system for space-based gravitational wave detection and demonstrated both its feasibility and its effectiveness in space-based applications [23]. Zou et al. improved the performance and security of continuous-variable quantum key distribution (CV-QKD) systems significantly through rigorous calibration of the BHD efficiency, thus providing critical theoretical and experimental support for CV-QKD technology [24].

Building on this foundation, our study extends BHD’s utility to gas sensing and measurement applications. We designed an MZI that was integrated with a BHD system, using its low noise, high SNR, and strong anti-interference capabilities to perform high-precision gas refractive index measurements. This approach overcomes the environmental sensitivity of the traditional methods discussed above to provide a robust solution for complex atmospheric and industrial monitoring scenarios. This proposed BHD-enhanced MZI operates on the principle that variations in the gas pressure induce refractive index changes, which then generate optical interference signals through the phase difference between the interferometer’s two arms. The proposed MZI uses a polarizing beam splitter (PBS) to split and combine the light and then applies the BHD method to detect the interference signal. Under conditions of a stabilized temperature of 25 °C, atmospheric pressure of 100.08 kPa, and relative humidity of 30%, the refractive index of air was measured experimentally to be n=1.0002711 with a minimum standard deviation of $1\times 10^{-7}$, thus demonstrating the high sensitivity and stability of the technique, and the advantages of noncontact measurement. This experimental result is closely aligned with the theoretical prediction of n=1.0002698 calculated via the Edlen formula [25,26]. The next section provides a detailed description of the experimental principle and its practical implementation.

## 2. Basic Principles

**Figure 1:**
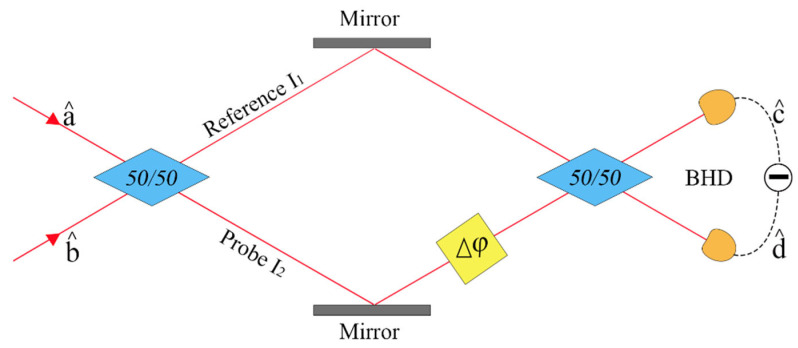
Free-space-type Mach–Zehnder interferometer (MZI).

A schematic diagram of a free-space-type MZI is shown in Figure 1. First, a 50/50 beam splitter divides the incident light into a probe beam and a reference beam. The probe beam then traverses the sample under test before being recombined with the reference beam to generate an interference signal, which can subsequently be detected using the BHD method. The experiment was designed based on this configuration.

When a coherent light state is injected into one port of the MZI and the other port is in a vacuum state, then based on the assumption that the phase change introduced by the sample is ∆φ, the emitted light fields can be expressed as follows:

(1) $$\hat{c}=\hat{a}sin\frac{∆\varphi }{2}+\hat{b}cos\frac{∆\varphi }{2}$$

(2) $$\hat{d}=\hat{a}cos\frac{∆\varphi }{2}-\hat{b}sin\frac{∆\varphi }{2}$$

After BHD, the difference between the photon numbers is given by:

(3) $$n_{dc}=\hat{d}\times \hat{d}-\hat{c}\times \hat{c}=\left(\hat{a}\times \hat{a}-\hat{b}\times \hat{b}\right)cos∆\varphi -\left(\hat{a}\times \hat{b}+\hat{b}\times \hat{a}\right)sin∆\varphi$$

where

(4) $$\langle n_{c}\rangle =\langle \hat{a}\times \hat{a}\rangle {sin}^{2}\frac{∆\varphi }{2}$$

(5) $$\langle n_{d}\rangle =\langle \hat{b}\times \hat{b}\rangle {cos}^{2}\frac{∆\varphi }{2}$$

The photon number fluctuations are then given by:

(6) $$∆n_{dc}=\sqrt{\langle n_{dc}^{2}\rangle -{\langle n_{dc}\rangle }^{2}}=\left|\alpha \right|$$

(7) $$n_{dc}=\langle n_{dc}\rangle +\langle n_{dc}\rangle dw\left(t\right)={\left|\alpha \right|}^{2}cos∆\varphi +\left|\alpha \right|dw\left(t\right)$$

where $dw\left(t\right)$ represents the Gaussian noise, and the normalization condition is $\langle dw\left(t\right)dw\left(\tau \right)\rangle =\delta \left(t-\tau \right){dt}^{2}$. The photocurrent in BHD can be expressed as follows:

(8) $$I\left(t\right)={\left|\alpha \right|}^{2}cos∆\varphi +\left|\alpha \right|dw\left(t\right)$$

where the first term ${\left|\alpha \right|}^{2}cos∆\varphi$ represents the phase-dependent interference signal, which corresponds to the classical intensity relationship $I_{s}=\frac{I_{1}}{2}+\frac{I_{2}}{2}\pm \sqrt{I_{1}I_{2}}\cos∆\varphi .$ In this relationship, $I_{1}$ and $I_{2}$ denote the optical intensities in the reference and sensing arms, respectively. The second term $\left|\alpha \right|dw\left(t\right)$ characterizes the quantum shot noise that arises from Poissonian photon statistics. The interferometric phase difference is given by ∆φ=2πδ/λ, where δ is the optical path difference and λ is the laser’s operating wavelength. When the probe arm senses changes in the refractive index or in the optical length in the light field, detectable phase shifts are induced through the cos∆φ term. These variations can then be detected via BHD, which enables phase-to-voltage conversion.

To measure the refractive index of a gas, a gas cell is placed in the probe arm. When the probe beam passes through the gas cell and the gas pressure varies, the optical path difference between the two arms of the interferometer also changes accordingly. The theoretical expression for the refractive index of the gas is derived as follows:

(9) $$n=k\frac{\lambda }{L}p+1$$

where $k=\frac{∆N}{∆p}$, ∆p is the pressure difference between the air chamber pressure and standard atmospheric pressure; ∆N denotes the number of interference fringes caused by the optical path difference; λ is the laser’s operating wavelength; and L is the effective interaction length of the laser with the gas.

## 3. Experimental Setup

**Figure 2:**
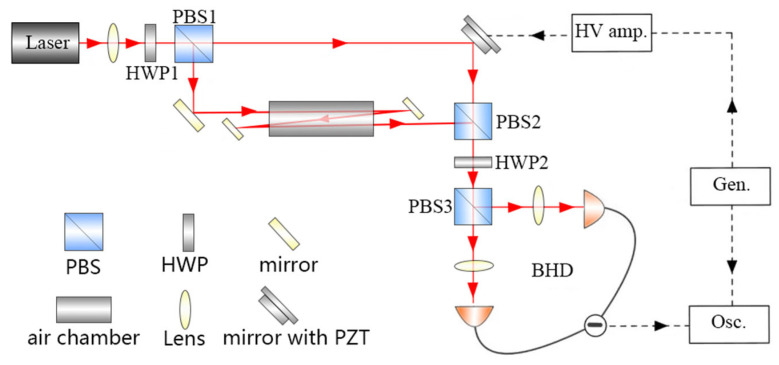
Experimental setup for the MZI. HWP: half-wave plate; PBS: polarizing beam splitter; PZT: piezoelectric ceramic element; Mirror with PZT: mirror with piezoelectric ceramic element; Gen.: signal generator, which applies a triangular wave-type scanning voltage to the PZT elements; HV amp.: high-voltage amplifier, which amplifies the triangular wave-type scanning voltage; Osc.: oscilloscope, which receives the detected signal from the BHD system.

The experimental interferometric system is illustrated schematically in Figure 2 (coupled with its corresponding Figure S1 in the accompanying Supplementary Materials). A continuous-wave narrow-linewidth Nd: YAG solid-state laser (∆v<10 kHz) with a central operating wavelength of 1064 nm outputs a collimated beam with a waist size of approximately 1 mm through a lens with a focal length of 150 cm. The laser light passes through PBS1 to be split into the reference beam $I_{1}$ and the probe beam $I_{2}$, and a half-wave plate (HWP1) is used to adjust the splitting ratio to 50/50. Reference beam $I_{1}$ is reflected by a mirror with a piezoelectric ceramic (PZT) element back toward PBS2; meanwhile, probe beam $I_{2}$ enters the gas cell and is then incident on PBS2, which combines the reference and probe beams. Following this, an optical interference signal is generated at HWP2. PBS3 is then combined with HWP2 to split the combined beam into two balanced interference outputs, where the polarization states are matched to achieve high interference visibility [27]. The PBS exhibits an extinction ratio of 30.5 dB and maintains polarization state stability over one-hour operation periods.

The gas cell is temperature stabilized with a temperature fluctuation of ±0.01 °C. The gas cell length is $L_{0}=80\;mm$. To increase the effective interaction length between the light and the gas, a folded optical path design is used that allows the laser beam to pass through the gas chamber three times to produce an effective length of ${L=3L}_{0}=240\;mm.$ The path length imbalance between the probe and reference arms (∆L ≈ 0.2 m) remains negligible when compared with the laser’s coherence length $(L_{c}>10^{4}$ m), which satisfies the interference condition $L_{c}\gg$ ∆L. The two balanced interference outputs are aligned precisely with the dual input ports of a BHD system, which consists of two identical photodiodes of the same model. The differential output is then digitized using a digital oscilloscope for signal acquisition. This polarization-stabilized architecture, which is composed of cascaded PBS-HWP elements, maintains a low polarization drift and long-term splitting ratio stability.

**Figure 3:**
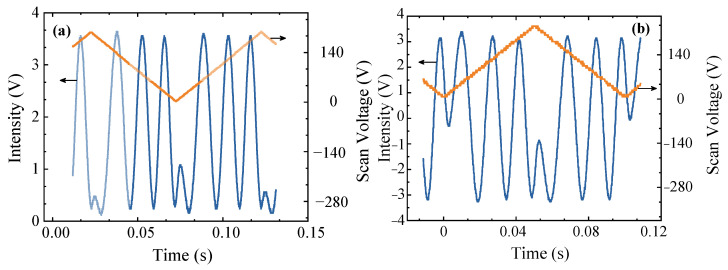
(**a**) Interference signal from one detection window of the BHD system unit; orange line: scanning voltage; blue line: interference signal. (**b**) BHD unit output.

Before the interferometric measurements can be performed, it is necessary to optimize and calibrate both the interference efficiency of the MZI and the common-mode rejection ratio of the BHD system. The signal generator outputs a triangular wave-type scanning signal with a frequency of 10 Hz and an amplitude of 6 V. This signal is then amplified using a high-voltage amplifier with a gain of 40 and is subsequently applied to the PZT to introduce periodic optical path difference changes. The resulting MZI interference signal is illustrated in Figure 3. Figure 3a shows the interference signal output by probe 1 in the BHD system, which has a peak-to-peak value of $V_{pp}=3.12$ V. The measured powers are $I_{1}$ = 1.68 mW and $I_{2}$ = 1.47 mW. The gain of the BHD unit is 1 V/mW. The theoretical peak-to-peak interference signal value$V_{PP}^{\prime }=2\sqrt{I_{1}I_{2}}$ can be calculated using the formula $I_{s}=\frac{I_{1}}{2}+\frac{I_{2}}{2}\pm \sqrt{I_{1}I_{2}}\cos∆\varphi .$ Therefore, the interference efficiency $\eta =V_{pp}/V_{PP}^{\prime }$. The interference efficiency of the MZI as measured using probe 1 is 99%. Similarly, the interference efficiency for probe 2 was measured to be 95%. The interference efficiency difference between these two detection windows is mainly caused by the extinction ratio difference in the PBS. The differential output interference signal from the BHD system has a peak-to-peak voltage of 6.84 V, as shown in Figure 3b. When compared with a single photodetector, the BHD system offers a superior SNR. Therefore, the differential interference signal from the BHD system is used for the measurements.

**Figure 4:**
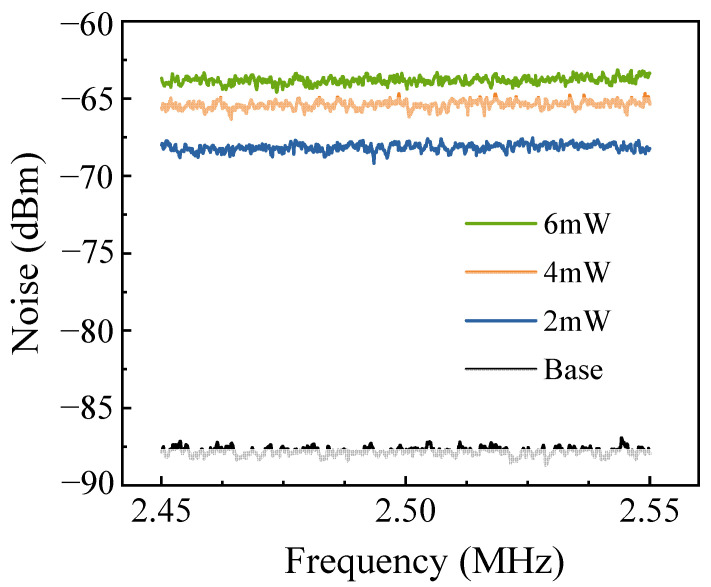
Noise power spectra of the BHD system output for various input light intensities.

The principal noise contributions that limit the sensing precision arise from the quantum noise in the input optical field and the electronic noise in the BHD system. To investigate these effects, the coherent light field intensity at the interferometer’s input was adjusted gradually, and the corresponding noise power spectrum after the BHD system was then measured. The results are presented in Figure 4. The measurements were conducted using a spectrum analyzer (N9000A, Keysight, Santa Rosa, CA, USA) with a resolution bandwidth (RBW) of 10 kHz and a video bandwidth (VBW) of 100 Hz. When no light was injected into the interferometer, the spectrum analyzer recorded an output noise level of −88 dBm, representing the electronic noise of the BHD system, or the background noise. Upon injection of laser light with a power of 2 mW into the interferometer, the noise power spectrum was measured again, showing a noise intensity of −68.2 dBm. This value is 19.8 dB higher than the background noise level. Further observations confirmed that the noise intensity increased synchronously with increasing input light power. Typically, the laser power injected into the interferometer must be more than 2 mW to realize a sufficient measurement SNR.

**Figure 5:**
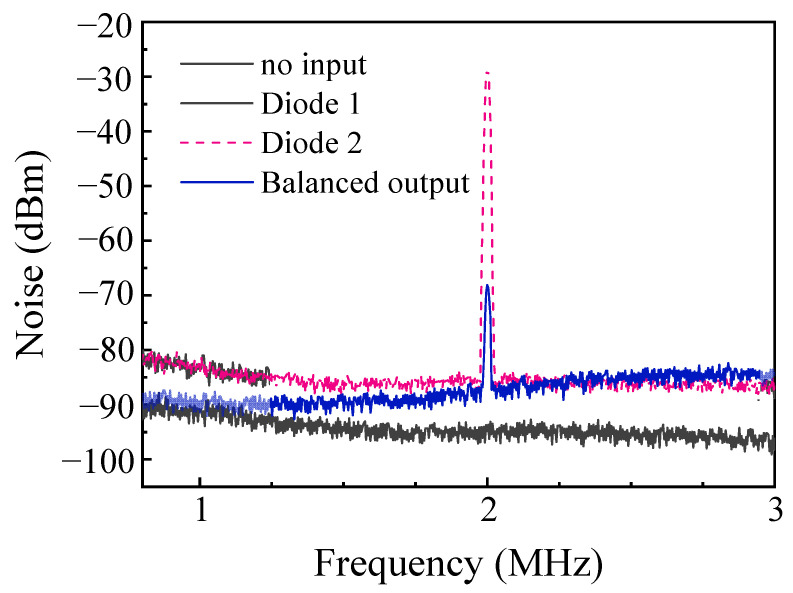
Measurement of the common-mode rejection ratio of the BHD system.

To characterize the noise suppression performance of our detection system, we measured the BHD system’s common-mode rejection ratio (CMRR) [28]. An electro-optic amplitude modulator was placed within one arm of the interferometer, while the other arm was blocked using a beam stop. A small-amplitude modulation at a frequency of 2 MHz was then applied to the modulator, enabling the BHD system to detect the modulated signal. As shown in Figure 5, the modulated signal detected within a single detection window of the BHD system was measured to be −27 dBm. Under the same experimental conditions, the modulated signal at the BHD system output was measured to be −67 dBm. The difference between these two signals indicates that the CMRR of the BHD system reaches 40 dB. This demonstrates the ability of the BHD system to suppress the common-mode noise when using the BHD technique.

## 4. Experimental Results

**Figure 6:**
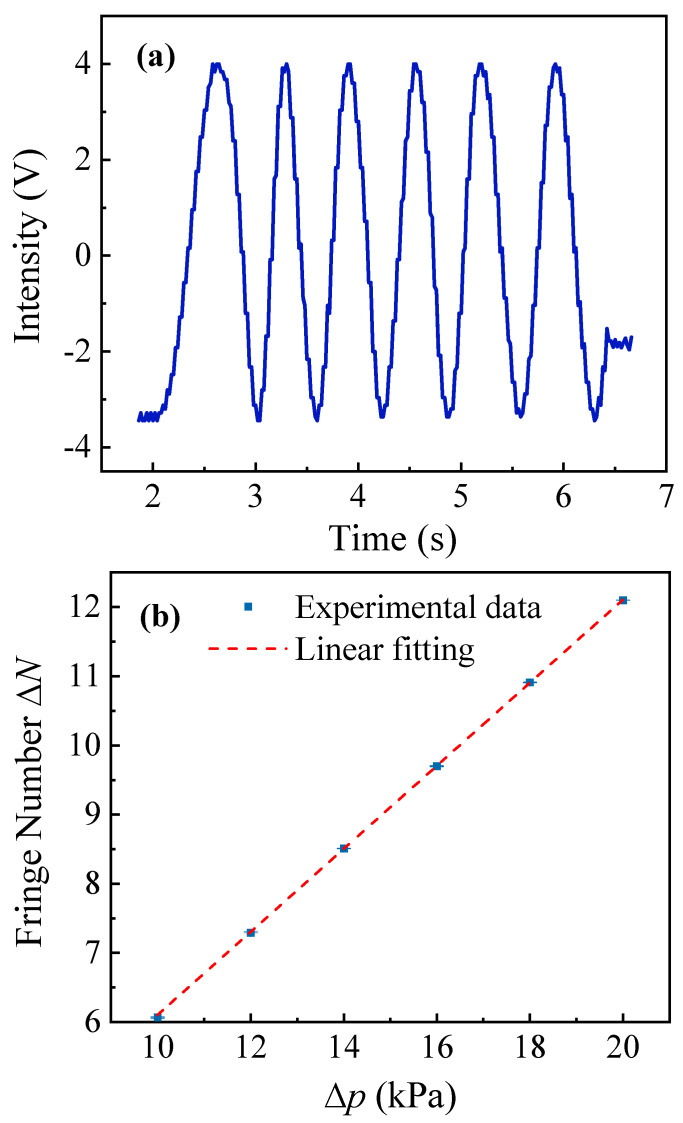
(**a**) Changes in interference intensity within the MZI during the gas release process. Here, ∆p was set at 10 kPa. (**b**) Number of interference fringes vs. gas cell pressure difference.

After the interferometer system’s performance was optimized, a gas cell with a length $L_{0}=80\;mm$ was placed in the probe arm of the MZI. This gas cell was connected to a pressure regulator to enable selection of a preset pressure that is monitored via a pressure gauge. The pressure gauge is Model ConST218A (ConST Instruments, China), which has a measurement range of 40 kPa and a long-term accuracy of 0.1% of the full scale. In the experiment, the probe beam traversed the gas cell three times via a folded optical path for a total interaction length of L=240 mm. Based on the sensing formula given as Equation (9), the sensing coefficient k is increased by a factor of three, which implies that the system’s sensitivity to changes in the pressure will also increase proportionally. Figure 6a illustrates the changes in the interference intensity within the MZI that occurred during the gas release process. The experimentally recorded parameters include the changes in the number of interference fringes ∆N, the pressure difference ∆p inside the gas cell, the atmospheric pressure of 100.08 kPa, the stabilized temperature of 25 °C, and a relative humidity of 30%. During the experiment, the interference signal was detected via a BHD system and recorded using a digital oscilloscope to enable post-processing. This measurement approach enabled precise determination of both the integer and fractional interference fringe counts. The integer part represents the total number of fringe cycles, and the fractional part is determined by substituting the corresponding amplitude value V obtained from the oscilloscope into the waveform function Acost=V, where A represents the amplitude of the interference signal. The fractional component was derived to be $\frac{t}{2\pi }$. This approach ensures precise quantification of the interference signal by integrating cycle counting with a waveform analysis. Five independent measurements of the fringe counts ∆N were conducted under varying values of the pressure differential ∆p, and the averaged values were subsequently analyzed. A linear fitting analysis indicated a proportional relationship between the fringe count and the pressure difference, as illustrated in Figure 6b. The relationship between ∆N and ∆p shows strong linearity.

**Figure 7:**
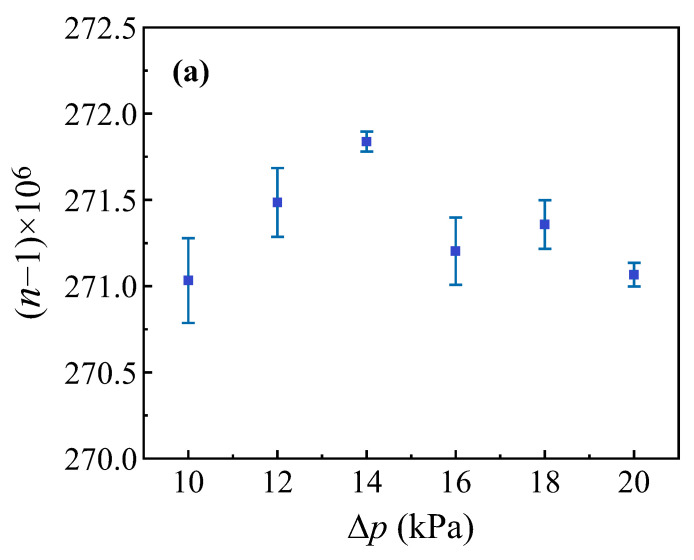

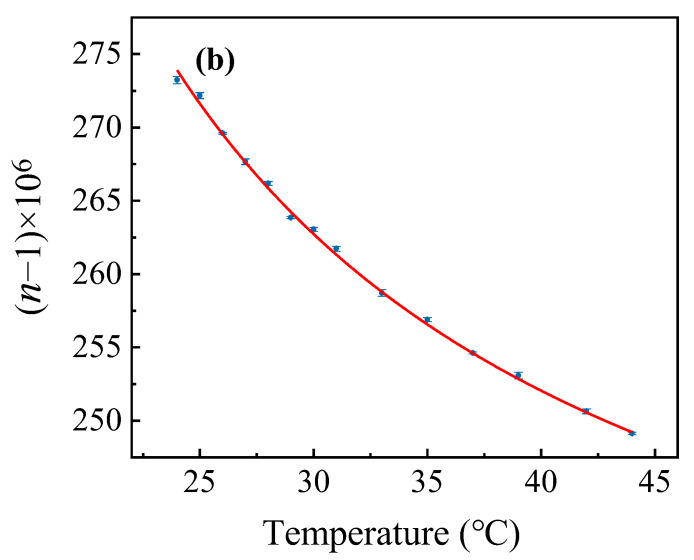
(**a**). Gas refractive index vs. gas cell pressure difference. The solid blue square represents the data points. The blue solid line represents the error bar. (**b**). Gas refractive index vs. temperature. The solid blue square represents the data points. The blue solid line represents the error bar and the solid red line represents the fitting curve.

Equation (9) can also be expressed in the form $n=\frac{∆N\cdot \lambda }{∆p\cdot L}p+1$. The pressure difference ∆p and the corresponding interference fringe counts ∆N obtained from the experiment can then be substituted into the equation to calculate the gas refractive index n. Data were collected five times for each pressure difference and the standard deviation of the measured values was then calculated. The measurement results are shown in Figure 7a, which illustrates the change in the refractive index of air in response to variations in the pressure differences in the gas cell. The measurements were taken at T=25 °C, p=100.08 kPa, and a relative humidity of 30%. The measured refractive index of air is n=1.0002711 with a minimum standard deviation of $1\times 10^{-7}$.

Next, we represent $n=\frac{∆N\cdot \lambda }{∆p\cdot L}p+1$ as $n=\frac{(N+\varepsilon )\cdot \lambda }{∆p\cdot L}p+1$, where N is the integer part of the number of interference fringes and ε is the fractional part of the number of interference fringes. Therefore, the combined measurement uncertainty of u(n) is given by:

(10) $$u\left(n\right)=\sqrt{u^{2}\left(∆p\right)+u^{2}\left(p\right)+u^{2}\left(\Delta T\right)+u^{2}\left(\varepsilon \right)+u^{2}(\lambda )+u^{2}\left(L\right)}$$

The first term results from the standard uncertainty of the pressure gauge. At the environmental temperature of 22 ± 0.5 °C, the short-term measured uncertainty is less than 0.004 kPa over a period of one hour, and its contribution to u(n) is $7.7\times 10^{-8}$. The second term reflects the impact of the atmospheric pressure. The fluctuation of atmospheric pressure in the cleanroom laboratory was maintained by an environmental control system operating at 22.0 ± 0.5 °C. Continuous environmental monitoring over a one-hour period revealed pressure fluctuations of 0.02 kPa. This variation contributes a relative standard uncertainty component $u\left(n\right)$ of $5.4\;\times 10^{-8}$ to our measurement system. The third term represents the effect of the temperature controller. The temperature fluctuation is ±0.01 °C and the influence of the temperature on $u\left(n\right)$ is $1\times 10^{-8}$. The fourth term represents the influence of the fringe resolution, which is measured by the BHD system with an uncertainty of $1.4\times 10^{-4}$, and its contribution $u\left(\varepsilon \right)$ is $4.4\times 10^{-9}$. The fifth term represents the error that results from the stability of the laser source, which has an uncertainty of $3.8\times {\;10}^{-8}$ µm, and this uncertainty contributes $9.6\times 10^{-12}$ to $u\left(\lambda \right)$. The sixth term represents the influence of material thermal expansion. Both sides of the chamber are made from quartz glass, which has a thermal expansion ratio of $5\times 10^{-7}\;{{}^{\circ}C}^{-1}$, and its contribution to $u\left(n\right)$ is $5.6\times 10^{-15}$. Finally, the root-sum-square combined standard uncertainty can be calculated to be $9.5\times 10^{-8}$. Experimental measurements show that the minimum standard deviation is $1\times 10^{-7}$, which is closely aligned with the theoretical prediction.

Furthermore, the pressure difference was maintained at 14 kPa, and the refractive index of air was measured within the temperature range from 24 °C to 44 °C. A fitting analysis was performed based on the Edlen formula [25,26] and the results are presented in Figure 7b. As the temperature increases, the refractive index of air is shown to decrease correspondingly. At a temperature of 25 °C with an atmospheric pressure of 100.08 kPa and a relative humidity of 30%, the refractive index of air n=1.0002611 was calculated using the theoretical formula. This calculation result is consistent with the reference value within the measurement error range. The method proposed in this study thus demonstrates high sensitivity and stability.

## 5. Conclusions

In this study, we demonstrate high-precision measurements of gas refractive indices using a free-space MZI integrated with a BHD system. Under controlled environmental conditions (25 °C, 100.08 kPa), the measured refractive index of air was determined to be *n* = 1.0002711 with a minimum standard deviation of $1\times 10^{-7}$, which was closely aligned with theoretical predictions. These experiments highlight the advantages of using the noncontact free-space MZI to perform precision measurements. When compared with traditional methods, the proposed approach improves the measurement sensitivity significantly. For example, unlike contact-based fiber optic sensing methods that are dependent on spectrometers, this noncontact scheme eliminates the resolution limitations of spectrometers and avoids the external interference introduced by contact measurements. Additionally, the interferometer exhibits minimal errors when reading interference fringes, and the BHD method achieves a CMRR of up to 40 dB, thus enhancing the SNR in the experiments significantly. This study provides an effective experimental approach for measurement of gas refractive indices, with potential applications in sensor technology, precision measurements, and related fields.

## Data Availability

No new data were created or analyzed in this study. Data sharing is not applicable to this article.

## Supplementary Materials

The following supporting information can be downloaded at: https://www.mdpi.com/article/10.3390/s25113519/s1, Figure S1: Experimental setup for the MZI. HWP: half-wave plate; PBS: polarizing beam splitter; PZT: piezoelectric ceramic element; Mirror with PZT: mirror with piezoelectric ceramic element; Gen.: signal generator, which applies a triangular wave-type scanning voltage to the PZT elements; HV amp.: high-voltage amplifier, which amplifies the triangular wave-type scanning voltage; Osc.: oscilloscope, which receives the detected signal from the BHD system.
