# Supplementary figures and images for: High-Precision Interferometric Measurements of Gas Refractive Index Using Homodyne Detection
Source: Sensors (Basel). 2025 Jun 3;25(11):3519. doi: 10.3390/s25113519 (PMC12158383; doi:10.3390/s25113519)

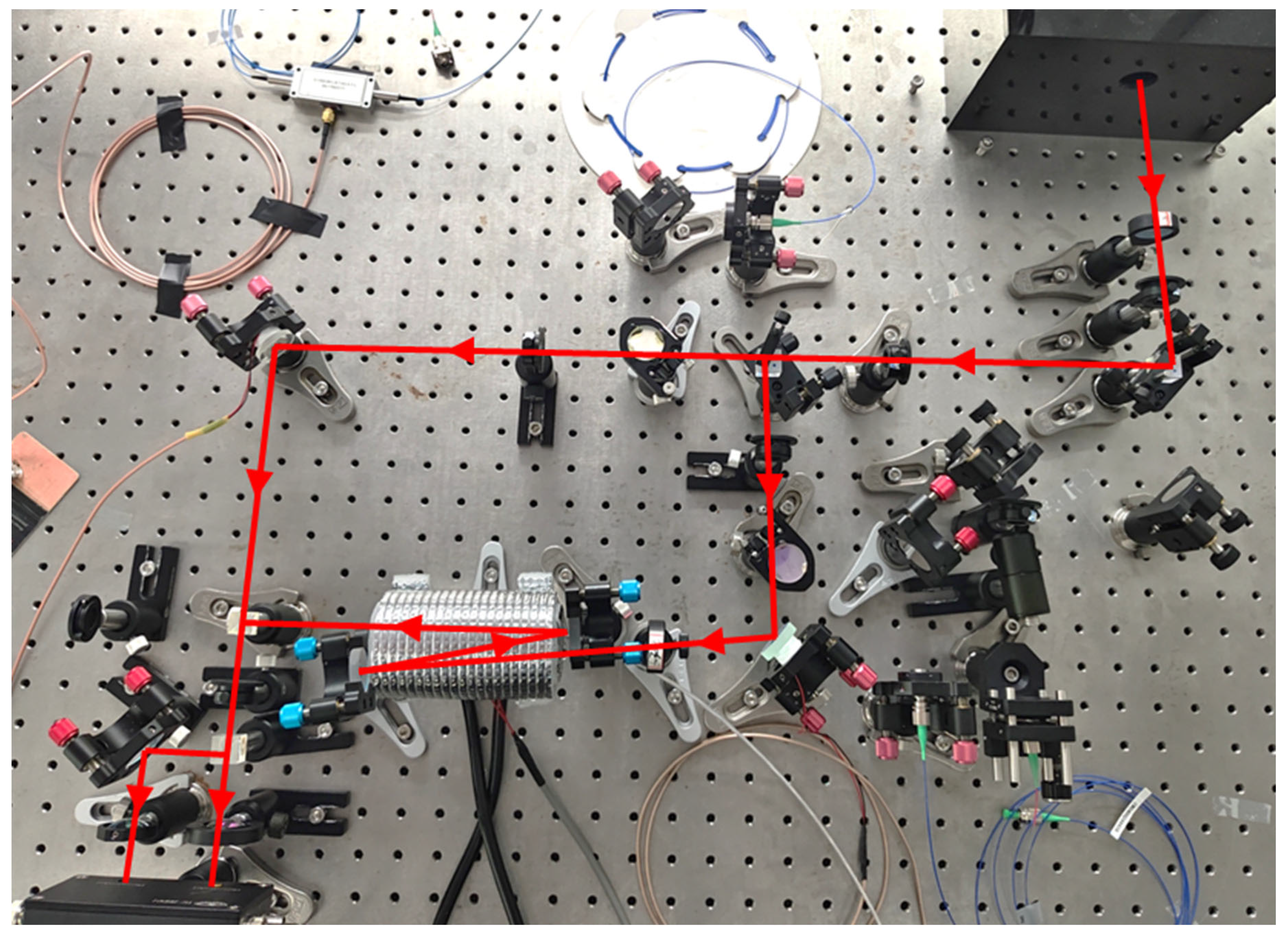

Supplement: Supplementary file 1 [file sensors-25-03519-s001.zip › sensors-3642091-supplementary.tif]
